# The 21^st^ Congress of the Academy for Multidisciplinary Neurotraumatology and the 3^rd^ Neurotrauma Treatment Simulation Center – Shifting the Paradigm in Neurotrauma Care

**DOI:** 10.25122/jml-2024-1010

**Published:** 2024-07

**Authors:** Stefana-Andrada Dobran, Alexandra Gherman, Dafin Fior Muresanu

**Affiliations:** 1 RoNeuro Institute for Neurological Research and Diagnostic, Cluj-Napoca, Romania; 2 Department of Neuroscience, Iuliu Hatieganu University of Medicine and Pharmacy, Cluj-Napoca, Romania

## THE ACADEMY FOR MULTIDISCIPLINARY NEUROTRAUMATOLOGY - Changing the Paradigm in Neurotrauma Care

The Academy for Multidisciplinary Neurotraumatology (AMN) strives to advance neurotraumatology in research, medical practice, and education, building upon three dimensions: organizing and participating in scientific events, supporting communication among national and international scientific entities, and committing to excellence in education by facilitating workshops and intensifying collaboration among scientific organizations.

The Academy is dedicated to fostering innovation and collaboration among specialists worldwide to ensure enhanced patient outcomes and improved quality of life. Through its efforts, the AMN aims to elevate the standard for neurotrauma care globally.

“Science and medical knowledge are always a snapshot; it can never capture the full truth. It's an ongoing process, and this applies also to scientific societies, like the AMN: it has to advance in order to stay a living entity. So, I’m excited that important developments at the AMN have come into life in recent years, also during my presidency here. Great educational endeavors, production of advanced international treatment guidelines, cutting-edge research implementing the multidimensional approach – it was so necessary to promote this. It's a living society, and I'm excited to be a part of this. And, in the end, this is also reflected by the change in the dynamic way the Academy outreaches the people, which, last but not least, is also expressed in the rebranding of the AMN.”

- Professor Johannes Vester

AMN President

With a community of over 600 members worldwide, specializing in neurology, neurosurgery, psychiatry, intensive medicine, and rehabilitation, among others, the AMN strives to create a network where specialists work jointly to address the most significant issues issues in neurotraumatology and find practical solutions, whether high-end options or limited resources are available. Through focused efforts, it encourages a new generation of experts to work, research, and educate in the spirit of multidisciplinarity when addressing one of the most complex and widely encountered neurological conditions - neurotrauma.

## 21 Years of Multidisciplinary Neurotraumatology

**Figure 1:**
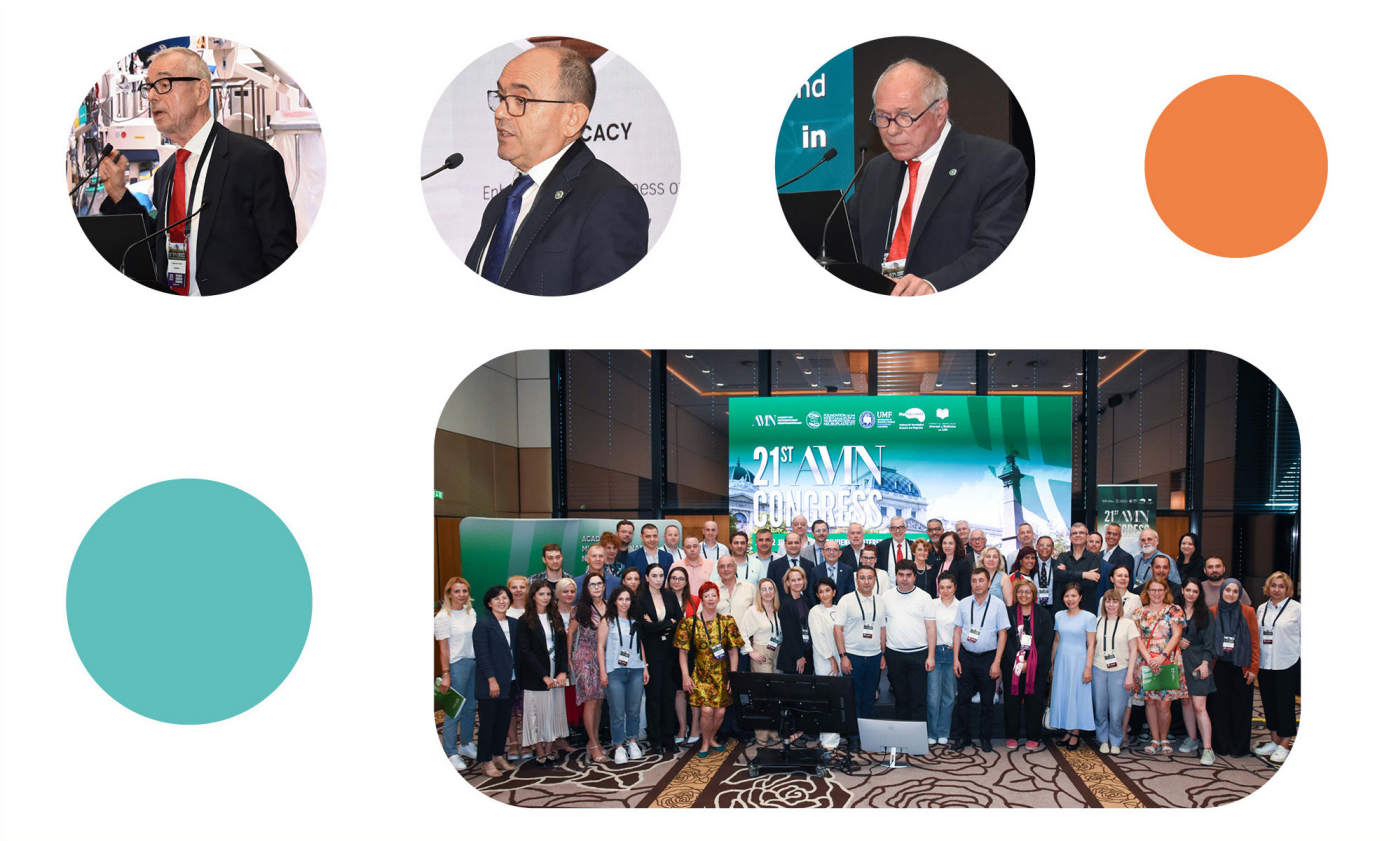
Prof. Johannes Vester, AMN President, Prof. Dr. Dafin Mureșanu, EFNR President, Prof. Dr. Volker Hömberg, WFNR President (top row, from left to right) and photo of the participants and speakers (bottom row)

The 21^st^ AMN Congress welcomed over 300 participants and internationally acclaimed speakers (Figure 1, Annex A) from several countries (e.g., Spain, Poland, Germany, Switzerland, the Netherlands, Slovenia, Austria, Romania, Turkey, Uzbekistan, Azerbaijan, Iran, Thailand, Vietnam, Taiwan, the Philippines, South Korea, Egypt, Canada, Mexico, Argentina) to address key subjects in neurotraumatology. The event took place on June 21-22, at the Hilton Vienna Waterfront, in Vienna, Austria. Upon this occasion, the Secretary General of the AMN, Prof. Dr Dafin Mureșanu stated a keynote message:

“The Academy for Multidisciplinary Neurotraumatology (AMN) is a pivotal force in the evolving landscape of neurotraumatology. In an era where true multidisciplinary collaboration is essential, AMN’s approach is unique as it merges cutting-edge research with global medical expertise, creating a platform that is indispensable for shaping the future of brain trauma care. By advancing both the theoretical and practical domains of neurotraumatology, AMN ensures that the insights of today become the innovations of tomorrow. Our vision for the future is clear - AMN stands as a beacon for transformative, patient-centric care in the field.”

**Figure 2:**
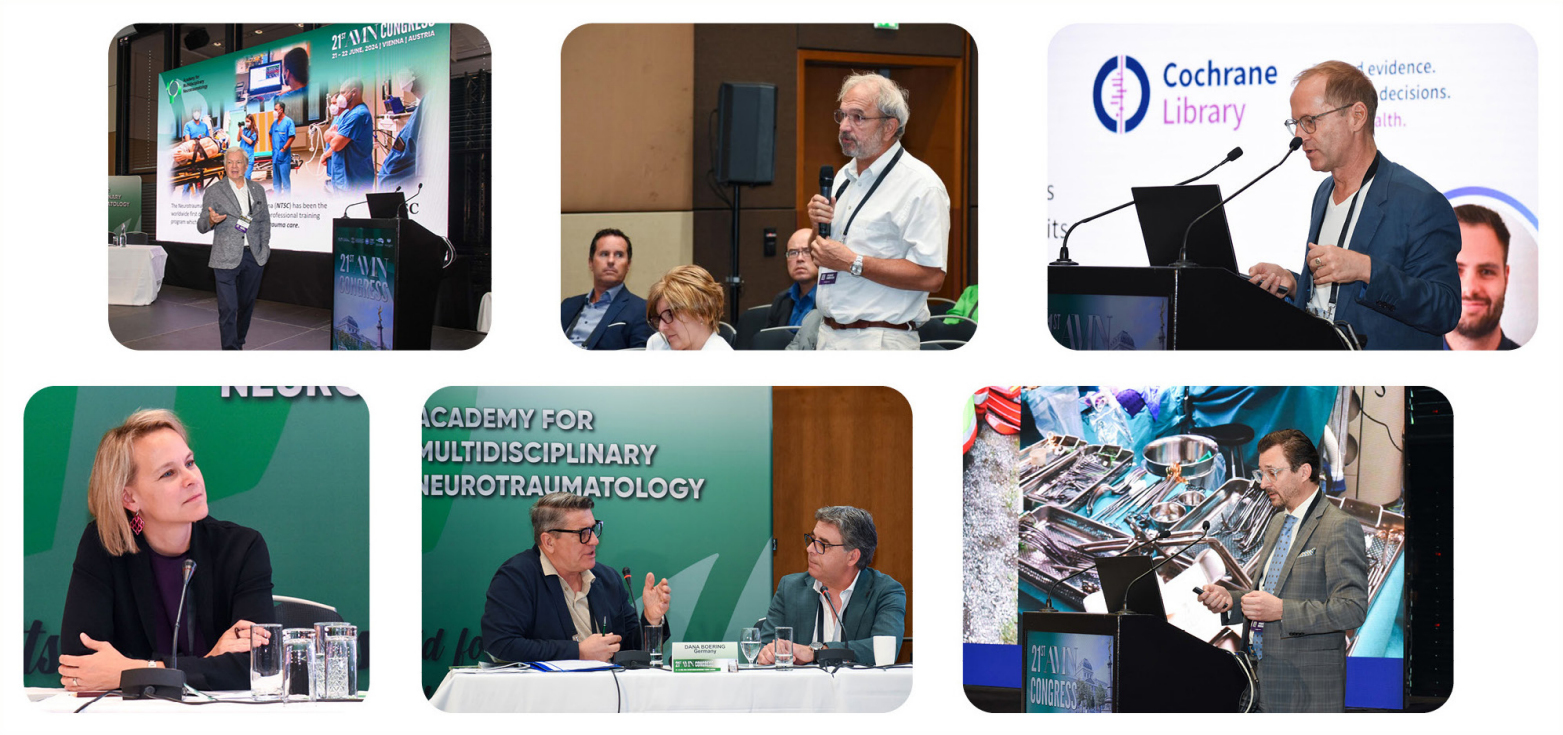
Speakers from the 21^st^ AMN Congress – Prof. Christian Matula, Prof. Helmut Trimmel, Prof. Peter Lackner (top row, from left to right); Dr. Vera Wohlgenannt, Dr. Andreas Winkler together with Prof. Antón Álvarez, and Prof. Harald Widhalm (bottom row, from left to right)

With support from the industry, over 40 speakers (Figure 2, Annex A), and dynamically constructed sessions, the Congress continued the long-standing tradition of promoting multidisciplinary perspectives, standing out through the comprehensive program that followed all aspects of the neurotrauma patient’s pathway through the medical system, from prehospital care to neurorehabilitation. Additionally, the program underlined the importance of neuropsychiatry and the development and implementation of comprehensive guidelines to ensure the best care for the patients.

The Congress featured the following activities and sessions:

- seven scientific sessions;
- two interview sessions;
- two training courses;
- two sessions focused on interactive case discussions;
- one presidential session;
- one multidisciplinary panel discussion;
- one ‘lunch & meet the experts’ session;
- one keynote lecture;
- one debate session.

Perspectives from high-end settings and innovations in resource-limited institutions converged to promote interactive knowledge exchange and mutual learning. The participants expanded their knowledge of high-tech equipment and complex treatment techniques, and gained a better understanding of how to ensure basic care and develop strategies to overcome financial, structural, or political limitations.

The program reflected the core principles of the AMN - changing the treatment paradigm from short-term focus to long-term follow-up in neurotrauma care in the spirit of multidisciplinarity.

**Figure 3:**
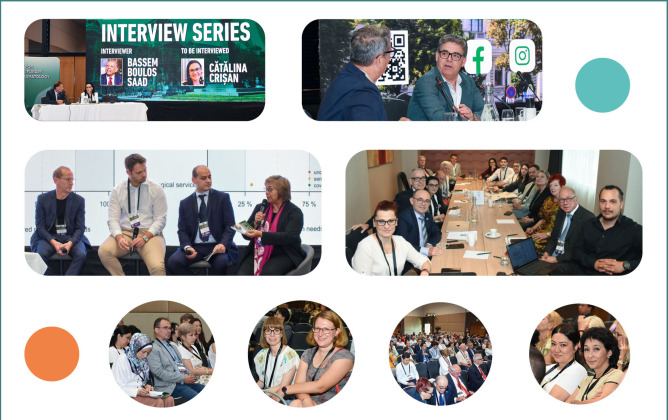
Photographs from the AMN Congress – the AMN Interview Series featuring Prof. Bassem Boulos Saad and Lecturer Cătălina Crișan (top left) and Dr. Andreas Winkler together with Prof. Antón Álvarez (top right); Panel discussion featuring, from left to right, Prof. Peter Lackner, Dr. Dominik Fortner, Prof. Felix Brehar, and Dr. Lynne Lucena (middle left); the AMN Board Meeting (middle right), and photos of the participants (bottom row)

The Congress featured an array of sessions, most of which approached key stages of the continuum of care for neurotrauma patients, from critical emergency interventions in the prehospital phase to hospital care and neuropsychological rehabilitation. Moreover, the training courses offered guidance on building successful intra-institutional neurotrauma teams and managing comprehensive neurocognitive post-TBI assessment. As a core part of the Congress, the presidential session emphasized the role and impact of the Academy within and on the entire academic spectrum, outlining its defining role in uniting international specialists from the clinical, pharmacological, and research fields. Lively debates engaged the audience on the subject of prehospital treatment organization, surgical and non-surgical approaches in acute treatment, and optimal timing for rehabilitation. At the end of the session, participants applied their knowledge using an audience response voting system. In addition, the TBI Interview Series facilitated knowledge exchange on experiences with neurotrauma care. Guidelines represented another focus, with presentations covering mild, moderate, and severe TBI, as well as surgical and non-surgical treatment. Real-life cases were explored during the clinical interactive case discussions, which included interactive quizzes that engaged the public. High-end hospital care was covered thoroughly, while a series of special lectures spotlighted the progress of the AMN in clinical research and that of the PRESENT (Patient REgistry – Short Essential NeuroTrauma) Registry. The comprehensive program (Figure 3) and its dynamic structure allowed for a well-rounded understanding of current practices and emerging innovations in the field.

During the AMN Congress, Prof. Dimitar Maslarov from the prestigious Medical University of Sofia launched his monograph. The book provides an accessible overview of all aspects of neurorehabilitation, from the latest technological advancements to pharmacological and non-pharmacological therapy. Written in an approachable manner, it emphasizes the importance of collaboration among specialists (e.g., neurologists, psychologists, nurses, and therapists) and underlines the need for personalized approaches in neurorehabilitation (Annex A).

The AMN Board Meeting held upon the occasion of the Congress brought together AMN and Neurotrauma Treatment Simulation Center (NTSC) core members, representatives of the European Federation of Neurorehabilitation Societies (EFNR) and the World Federation for Neurorehabilitation (WFNR) as well as guests from the industry, medical, and public health fields. The central aim of the discussions was to create a large and internationally acknowledged Academy through:

- Connecting AMN with other internationally recognized medical and scientific societies;
- Further developing joint activities and programs as well as hands-on sessions and intensive training courses;
- Consolidating the main pillars of the AMN (education, clinical practice, research and innovation, advocacy);
- Expanding NTSC at the international level;
- Advancing the PRESENT registry.

The program of the event and the list of speakers is available on the AMN website.

The AMN maintains partnerships with significant international stakeholders, such as the EFNR, WFNR, Foundation of the Society for the Study of Neuroprotection and Neuroplasticity (SSNN), the RoNeuro Institute for Neurological Research and Diagnostic, the European Academy of Neurology (EAN), the European Society of Clinical Neuropharmacology (ESCNP), and others, which further reflects the international dimension and expansion of the Academy and its dedication to advancing global standards in neurotrauma care.

## NEUROTRAUMA TREATMENT SIMULATION CENTER - A PATH TOWARDS MULTIDISCIPLINARY NEUROTRAUMA CARE

The Neurotrauma Treatment Simulation Center, now at its third edition, continued to train specialists with backgrounds in neurology, neurosurgery, anesthesiology, trauma medicine, and more, in the spirit of multidisciplinary teamwork. This initiative brings together participants from diverse countries to improve the quality of care for patients with neurotrauma. This year’s event welcomed delegations from Azerbaijan, Iran, Poland, Taiwan, Uzbekistan, Vietnam, and the Philippines, from 17-20 June in Vienna, Austria.

“I'm very proud that in particular the NTSC – the Neurotrauma Simulation Center Vienna – is such a wonderful project and is so successful [...]. Interestingly, how all that started is very easy to explain: a group of people came together and we were thinking [about] how we can make our job much better, and the shift in paradigm is how we were looking at that subject. So, the big difference is that we are looking at the full frame of neurotrauma care, [which] means from [the] unseen [of] where the trauma has happened, over all those different stations, until the people are discharged back into the community and social lives [...]. And that was the birth of the Neurotrauma Simulation Center — and simulation, therefore, because it's very important to train all those things based on a simulation program.”

– Professor Christian Matula,

AMN Chair of the Educational and Training Committee

**Figure 4:**
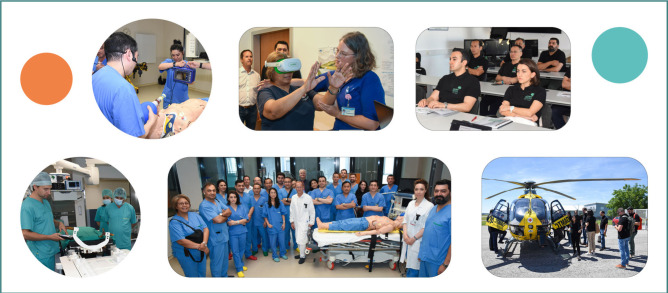
Photos at the leading Institutions from the Neurotrauma Treatment Simulation Center: Klinik Floridsdorf (top left and bottom center), AUVA Rehabilitationszentrum Meidling (top center), Landesklinikum Wiener Neustadt (top right and bottom right), and Allgemeines Krankenhaus Wien (bottom left)

Similarly to the last years [1, 2], during the four days of training (Figure 4), participants explored the patient’s pathway to recovery, starting from fundamental principles of emergency medicine at Landesklinikum Wiener Neustadt, where Prof. Trimmel and his colleagues discussed the procedures for medical emergencies in Austria, the roles of the rescue team members, and delved into the specifics of air rescue, for which the institution hosts an operational base. Later on the same day, the participants got acquainted with the development and objectives of the NTSC and AMN. On the second day, simulation exercises trained and engaged the participants in interdisciplinary treatment concepts, in one of the most advanced simulation centers located at Klinik Floridsdorf, under the guidance of Prof. Peter Lackner and his team. The scenarios model real-life emergencies and require participants to collaborate under time-sensitive conditions, offering an immersive experience on neurotrauma management. At the Allgemeines Krankenhaus Wien (AKH), Profs. Christian Matula, Johannes Leitgeb, Harald Widhalm, and their team educated the participants on interdisciplinary neurotrauma management and the distinct roles of specialists in the chain of neurotrauma, from the neurosurgeon to the traumatologist and anesthesiologist. Moreover, the attendees learned how to build a neurotrauma program and team. The last part of the patient's pathway brought the participants to AUVA Rehabilitationszentrum Meidling, where Drs. Vera Wohlgenannt and Andreas Winkler, along with their ccolleagues, outlined the specifics of TBI rehabilitation in Austria, including rehabilitation in the early stages of TBI, neuropsychological rehabilitation, physiotherapy, and occupational therapy, and introduced the participants to the latest techniques and equipment available on-site. Noteworthy, on all days, participants toured the institutions to see firsthand the resources and technology used in the specialized units and learn about treatment protocols.

The program offered an immersive experience in multidisciplinary neurotrauma management, incorporating essential aspects of emergency medicine, neurosurgery, trauma surgery, neurology, and rehabilitation. Prof. Christian Matula (Professor of Neurosurgery at AKH and AMN Chairman of the Educational Committee), Assoc. Prof. Johannes Leitgeb (University Clinic for Trauma Surgery at AKH), Assoc. Prof. Harald Widhalm (Department of Orthopedics and Traumatology at AKH), Dr. Andreas Winkler (Medical Director of the Institute Neuromed, Center for Clinic Sciences in Neurology), Dr. Vera Wohlgenannt (Deputy Medical Director at AUVA Rehabilitationszentrum Meidling), Prof. Helmut Trimmel (Director of the Department Anaesthesiology, Emergency, and Critical Care Medicine at Landesklinikum Wiener Neustadt), Prof. Peter Lackner (Head of the Department of Neurology at Klinik Floridsdorf), Drs. Daniel Csomor and Günther Herzer (Landesklinikum Wiener Neustadt) and their colleagues played a central role in the training program.

The implementation plans (Annex A), a core feature of the program, involve participants from each country setting up strategies to apply the lessons learned during NTSC to their institutions. This ensures active engagement and long-term outcomes, as the Academy actively collaborates with participants from earlier editions.

Interviews with participants and faculty (Annex A) explored how the program supports personal development, teamwork, and collaboration. Moreover, they highlighted future hopes for the program, the AMN’s role in multidisciplinary neurotrauma management, and the skills and development opportunities that the NTSC provides. A series of testimonials offered valuable insights from participants on the role of their background within the learning process and the lessons gained throughout the week. The interviews will be available on the AMN Blog and YouTube channel.

“The neurotrauma treatment chain is as strong as its weakest link.”

- Professor Christian Matula, AKH

As the NTSC evolves, it aims to set an international standard for training in the management of neurotrauma and inspire professionals to combine their creativity, expertise, and courage to shift the paradigm in neurotraumatology!

## CONCLUSIONS

Research, education, and medical practice are crucial for advancing neurotraumatology. Each patient's journey is unique; therefore, only through combined efforts and collaboration can the ultimate goal be achieved — to improve the quality of care at all levels, from emergency medicine to rehabilitation, for patients affected by neurotrauma.

**Figure 5:**
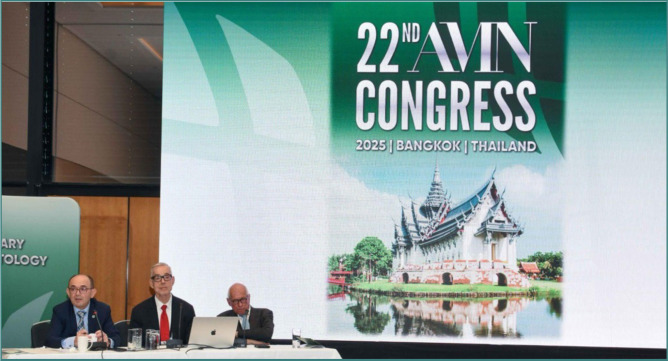
Prof. Dafin Mureșanu, Prof. Johannes Vester, and Prof. Volker Hömberg (from left to right) announcing the 2025 AMN Congress in Bangkok, Thailand

We welcome specialists worldwide to attend the next year’s Congress (Figure 5) and become members of our growing community - together, we can shape the future of neurotraumatology!
